# Supplementary material for: Long-Term Treatment with Gadopentetic Acid or Gadodiamide Increases TRPC5 Expression and Decreases Adriamycin Nuclear Accumulation in Breast Cancer Cells
Source: Cells. 2023 May 3;12(9):1304. doi: 10.3390/cells12091304 (PMC10177392; doi:10.3390/cells12091304)
Supplement: Supplementary file 1 [file cells-12-01304-s001.zip › cells-2211648-supplementary.pdf]

Supplementary figures

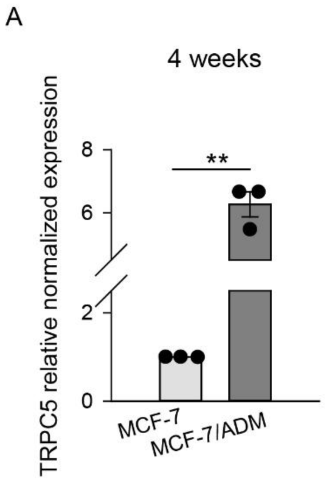

Figure S1. qRT-PCR analysis showed that TRPC5 expression was greater in MCF-7/ADM compared to MCF-7 cells. $**P < 0.01$.

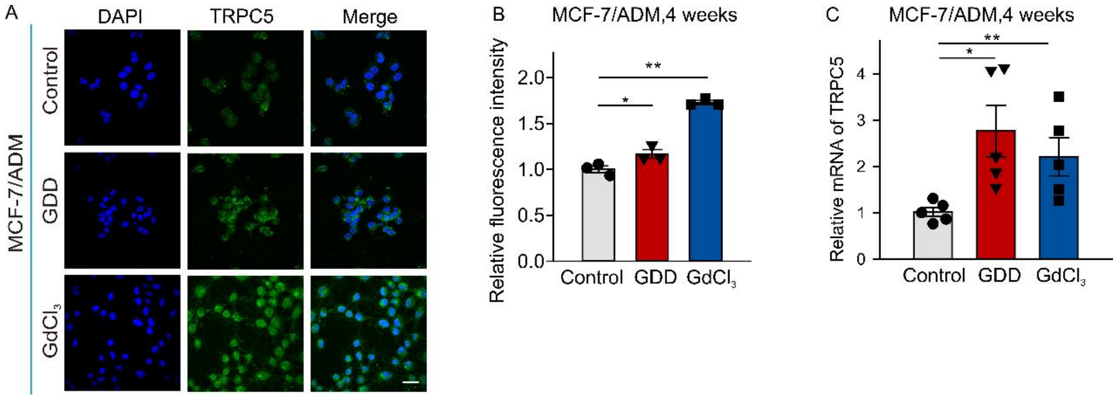

Figure S2. TRPC5 expression was greater in MCF-7/ADM treated with GDD and $GdCl_3$ for 4 weeks. **A and B** Representative images of immunostaining with TRPC5 and summary data (n=4) in MCF-7/ADM cells (TRPC5, green fluorescence). **C** qRT-PCR analysis of TRCP5 expression in MCF-7/ADM cells (n=5). The One-way ANOVA test followed by the Student-Newman-Keuls post hoc all pair-wise multiple comparison test was used to compare the treatment groups with the control group. All values are mean ± SEMs. $*P < 0.05$, $**P < 0.01$. The scale bar indicates 30 μm.

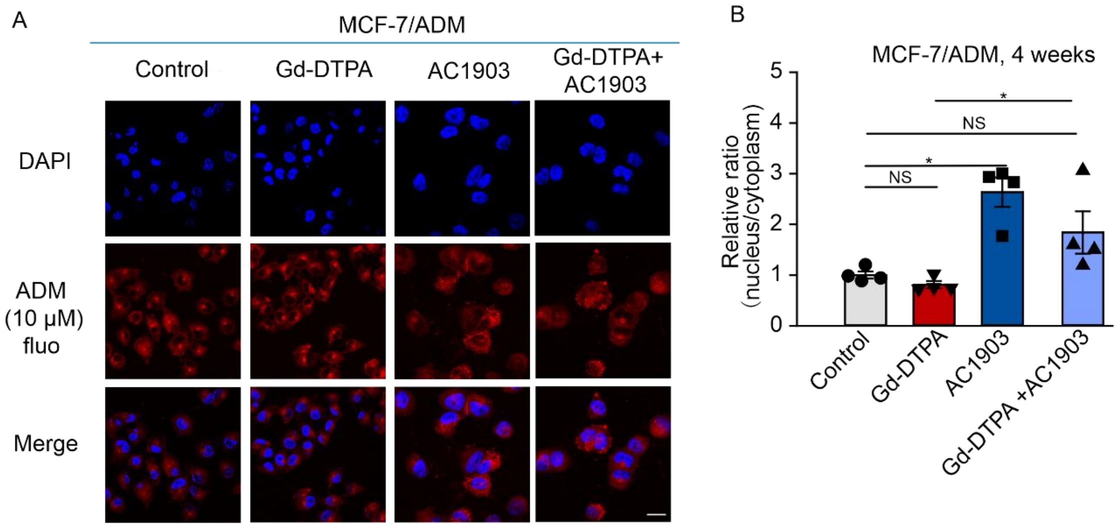

Figure S3. Downregulation of TRPC5 expression resulted in a decreased efflux of ADM in MCF-7/ADM cells. **A** Nuclear ADM accumulation in MCF-7/ADM cells. Representative confocal micrographs of MCF-7/ADM cells are shown. ADM exhibits red autofluorescence. The scale bar indicates 30 μm. **B** Summary data of ADM accumulation in MCF-7/ADM cells (n=4). The One-way ANOVA test followed by the Student-Newman-Keuls post hoc all pair-wise multiple comparison test was used to compare the treatment groups with the control group. All values are mean ± SEMs. *P < 0.05, **P < 0.01. "NS" stands for no significant difference.
